# Long-term neurostructural and psychological effects of war stress in two generations of civilians from the former Yugoslavia

**DOI:** 10.1038/s41598-026-44241-w

**Published:** 2026-03-17

**Authors:** Monika Fňašková, Pavel Říha, David Ulčák, Marek Preiss, Markéta Nečasová, Nikola Wolframová, Vojtěch Svoboda, Martin Lamoš, Ivan Rektor

**Affiliations:** 1 Centre for Neuroscience, Central European Institute of Technology (CEITEC), Masaryk University, Kamenice 5, 625 00 Brno, Czech Republic; 2 National Institute of Mental Health, Topolova 748, 250 67 Klecany, Czech Republic; 3 First Department of Neurology, Faculty of Medicine, St Anne’s University Hospital, Masaryk University, Brno, Czech Republic; 4 University of New York in Prague, Londynska 41, 120 00 Prague, Czech Republic; 5 Utrecht University, Heidelberglaan 8, 3584 CS Utrecht, The Netherlands

**Keywords:** MRI, Stress, Civilians, War, Crus II, Posttraumatic growth, Neuroscience, Psychology, Psychology

## Abstract

The long-term neurobiological and psychological effects of war-related stress on civilians remain understudied. This study focuses on survivors of the war in the former Yugoslavia (G1) who now reside in the Czech Republic and on the children of survivors (G2) who were born after the conflict. Participants from G1, G2, and a control group (CG) with no war experience underwent structural MRI, answered a semi-structured interview, and completed psychological questionnaires (PTGI, PCL-5, SWSL, MSPSS, Brief-COPE). Voxel-based morphometry was used to assess brain volume differences. Compared to CG, G1 showed reduced grey matter volume in regions associated with PTSD and autobiographical memory, including Crus II, parahippocampal gyrus, middle temporal gyrus, and fusiform gyrus. Psychologically, G1 reported higher PTSD symptoms, lower life satisfaction, and greater post-traumatic growth. G2 showed no structural brain changes but scored higher on post-traumatic growth than CG, with no significant differences in other psychological measures. The findings suggest long-lasting neuroanatomical and psychological effects of war stress in directly exposed individuals (G1). Although G2 showed no brain alterations, the increased post-traumatic growth may indicate subtle adaptation effects of growing up in a post-war environment, but not necessarily only in a maladaptive sense.

**Supplementary Information:**

The online version contains supplementary material available at 10.1038/s41598-026-44241-w.

## Introduction

The conflict in the former Yugoslavia, which lasted from the 1990s to the beginning of the new millennium, was one of the worst and most extensive in Europe since World War II. The wars that successively affected Slovenia, Croatia, Bosnia and Herzegovina, and Kosovo beginning in 1991, and the NATO bombing of Serbia and Montenegro in 1999 in response to the conflict in Kosovo, left the population affected by many traumatic experiences including war injuries, death of loved ones, separation of families, involuntary displacement from their homes, sexual violence, and torture^1,2^. Post-war development in these countries devastated by war or bombing has been difficult. Psychological help has not been routinely available, and untreated trauma has often manifested itself in alcohol abuse or violence^3^. The impact of poverty and harsh conditions, and the daily narrative of the war, has taken a toll on the next generation^4,5^.

This study investigates the long-term effects of war-related stress on civilian populations (G1) and the subsequent generation (G2), who are descendants of war survivors from the countries of the former Yugoslavia, though not directly related to the G1 participants. Utilising structural magnetic resonance imaging, a psychological survey, and interviews, we explore whether and how the impacts of war have influenced their current lives.

The psychological consequences of war include depressive and other mood disorders, anxiety, paranoid ideations, and post-traumatic stress disorder (PTSD)^6–8^. PTSD is generally considered an illness caused by a traumatic experience. It has been reported that in the general population, it arises as a result of trauma in about 5–10% of cases. In the case of war-related stress, the incidence approximately doubles, and as many as 26% of individuals who have remained in war-afflicted regions have been found to suffer from the disorder^9^. This trauma-related psychopathology represents a severe mental health condition that substantially compromises individual functioning and psychosocial well-being. Symptom clusters commonly include intrusive recollections such as flashbacks and distressing thoughts, avoidance of trauma-related stimuli, and hyperarousal. The traumatic experience becomes integrated into the individual’s identity and fundamentally alters self-concept and interpersonal functioning^10^.

Neuroimaging studies investigating the effects of traumatic psychological stress have consistently demonstrated alterations in brain structure and function, including reduced grey matter volume, often associated with the development of PTSD^11–15^. Although the hippocampus, amygdala, and prefrontal cortex are commonly identified as the primary stress-sensitive structures, many other regions undergo structural or functional changes due to stress. In particular, brain regions such as the insula and the anterior cingulate cortex (ACC) are frequently implicated in relation to stress and trauma^16,17^. In addition to the timing, intensity, and duration of psychological stress, one of the main factors affecting the brain appears to be the nature of the traumatic experience^18^. Studies dealing with the effect of war trauma on the alteration of brain structures in non-military personnel are scarce; most studies have been conducted on soldiers or war veterans^19,20^. Even though research on civilians is more demanding due to the high individual variability in stress responses (influenced by factors such as geographical proximity to conflict zones, duration of conflict, varying degrees of psychological vulnerability, etc.), non-existent or inadequate prior medical records, financial demands of research or non-existent research facilities in a war-torn country, and many other reasons, it is an important topic that should be given adequate attention, because unexplored and untreated trauma may have far-reaching effects on individuals and entire societies.

In the context of the war in the former Yugoslavia, we found only one MRI paper focused on brain structure volumes conducted on a group of Croatian war veterans who were not professional soldiers. The authors found a significant reduction in right hippocampal volume and a non-significant reduction in left hippocampal volume in this group^21^.

The next part of our research focuses on the descendants of the survivors - the second generation (G2). Interest in researching the effects of war on the next generation was sparked by vague but consistent symptoms in the descendants of Holocaust survivors^22–25^. With further studies, transgenerational transmission became a proven phenomenon, when it was shown that the stress experienced by parents can also affect offspring who were not directly exposed to trauma^26^. The mechanism of transgenerational transmission can occur at several levels (social transmission, epigenetic, prenatal) or their combinations^27^. Unlike animal models, where it is possible to control the conditions of the research^28^, human studies offer only very limited possibilities to determine how and in whom transmission can occur. A study of Kosovar families 11 years after the war found a connection between paternal PTSD, anxiety and depressive symptoms, and depressive symptoms in offspring^29^. In our current work, we must take into account the possible influence of an unfavourable post-war environment. ^29^

Although the conflict in the former Yugoslavia affected the lives of millions of people, little attention has been paid to it, at least in terms of neuroimaging. The aim of our study is to test whether the impact of the war is detectable after almost three decades, whether and how it affects survivors, and whether it has been transmitted to the next generation growing up in an adverse post-war environment.

Based on previous research and the aims of the present study, we hypothesize that individuals from the first generation exposed to war-related trauma will exhibit both neurostructural alterations, specifically reduced grey matter volume in brain regions implicated in stress and trauma processing, and persistent psychological impacts such as elevated PTSD symptoms. Furthermore, we expect that the second generation (G2), although not directly exposed to trauma, will show signs of transgenerational transmission of trauma effects, manifested as psychological alterations influenced by their parents’ war experiences and the adverse post-war environment.

## Results

### MRI

**Fig. 1:**
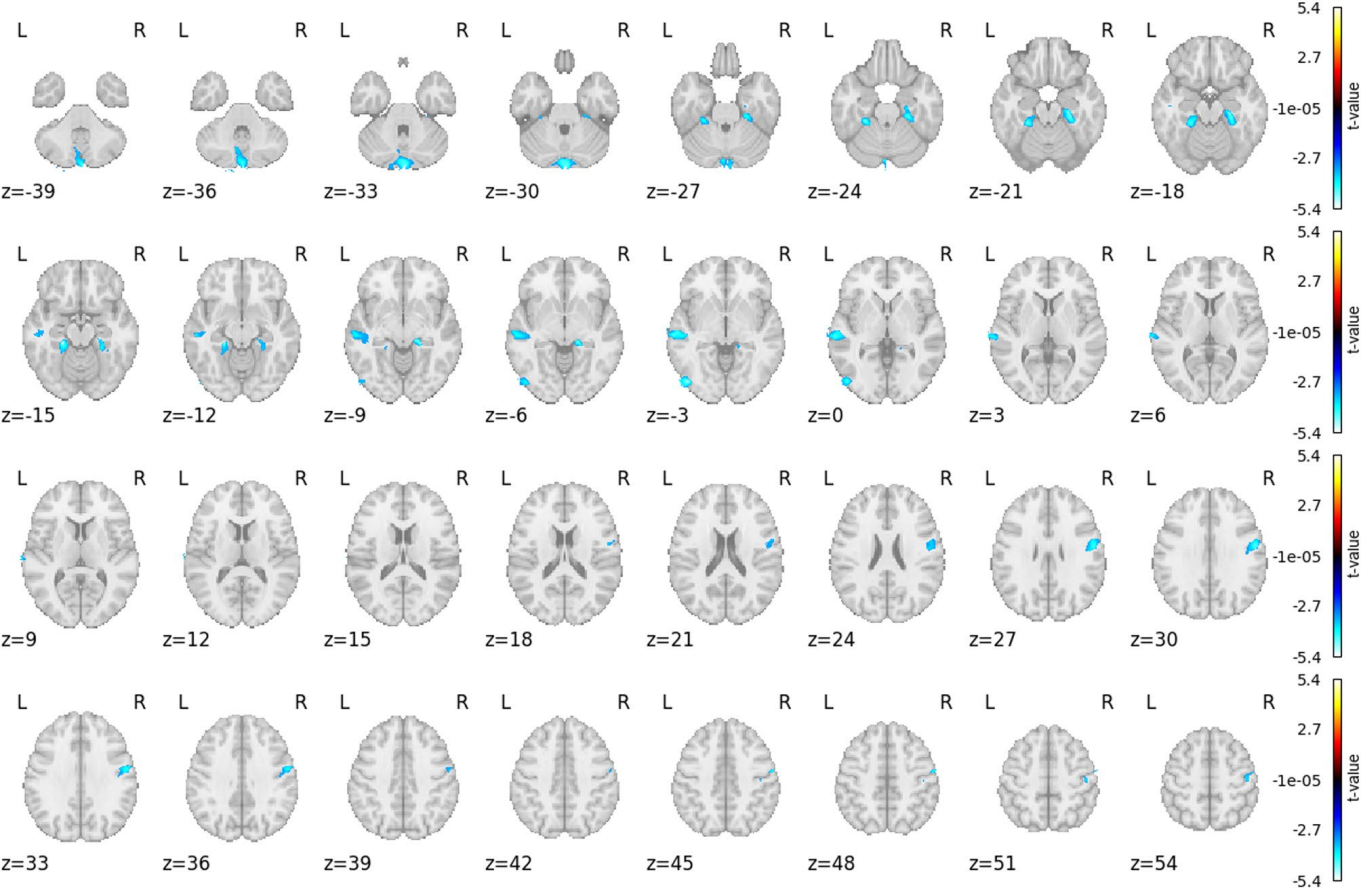
Comparison of grey matter volume between survivors (G1 group) and relevant control group.

Voxel-based morphometry (VBM) revealed a significant GM volume reduction in G1 as compared to control group relevant to G1 (CG1), as shown in Fig. 1; Table 1.

**Table 1:** Six significant clusters after correction (on each row), where the grey matter volume in G1 is smaller than in CG1.

Laterality	Structures	*p*-corrected	Peak (MNI, mm)	Cluster size (cm^3^)
L	Fusiform ParaHippocampal Cerebellum 4 5	0.0334	-21.5;-30.5;-14.5	1.986
	Temporal Mid	0.0045	-63.5;-21.5;-2.5	3.267
	Occipital Inf	0.0018	-46.5;-79.5;-3.5	1.093
L and R	Cerebellum Crus2	< 0.0001	1.5;-89.5;-31.5	4.22
R	ParaHippocampal Fusiform	0.0015	16.5;-29.5;-7.5	2.802
	Postcentral Precentral	0.0026	54.5;-5.5;29.5	3.054

The G2 group does not exhibit any structural changes based on the conducted GMV analysis compared to the control group.

### Psychological questionnaires

The G1 and CG1 groups, as well as the G2 and CG2 groups, were each compared separately based on their responses to psychological questionnaires. In the G1 vs. CG1 comparison, significant differences were found in the PTGI, PCL-5, and SWSL questionnaires, with G1 scoring higher across all measures. In the G2 vs. CG2 comparison, a significant difference was observed only in the PTGI questionnaire, where G2 scored higher than CG2. The results of both comparisons are presented in Table 2.

**Table 2:** Comparison of psychological questionnaire results between survivors (G1 group) and their control group (CG1), and between children of survivors (G2 group) and their control group (CG2).

Variable	G1 Median (IQR)	CG1 Median (IQR)	*p*-value
PTGI	56 (18)	39 (26)	**< 0.001**
PCL-5	19 (16)	13 (20)	**0.009**
SWLS	23 (8)	26 (9)	**0.040**
Brief COPE	67 (12)	66 (10)	0.930
MSPSS	67 (20)	69 (16)	0.4670

Variable	G2 Median (IQR)	CG2 Median (IQR)	*p*-value
PTGI	59 (30)	39 (25)	**0.004**
PCL-5	19 (17)	20 (25)	0.673
SWLS	23 (10)	26 (7)	0.151
Brief COPE	70 (14)	70 (13)	0.693
MSPSS	72 (15)	70 (12)	0.440

### Self-report and interview questions

#### Survivors of conflicts in the former Yugoslavia (G1 group)

**Fig. 2:**
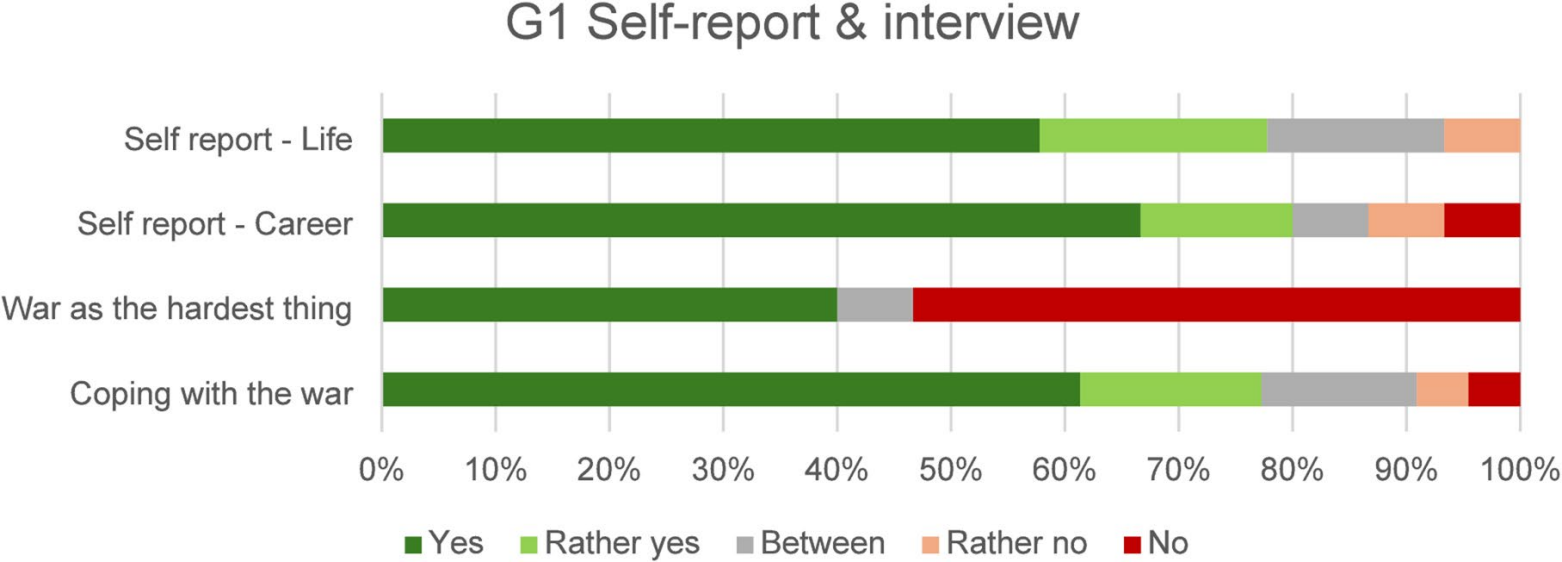
G1 responses in the self-report questionnaire and to interview questions.

In a self-assessment questionnaire, participants rated their satisfaction with their personal and professional lives so far. Overall, they expressed a high level of satisfaction in both areas: 78% of participants were satisfied with their personal lives (Self-report - life) and 80% with their careers (Self-report - career); the results are shown in Fig. 2. For the interview question *Was the war the hardest thing you have ever experienced in your life?*, 53% said no, and 40% said yes. For the question *Do you think you coped well with the war?*, 77% said yes; however, some elaborated on their experiences with specific triggers that induce a sense of threat. These triggers include the sounds of sirens, ambulances, planes, and similar noises. Additionally, many emphasised that the feeling of threat was heightened by the outbreak of Russian aggression against Ukraine in 2022 and by the uncertainty that accompanied the onset of the Covid-19 pandemic in 2020. The answers are shown in Fig. 2 (*War as the hardest thing*, *Coping with the war*).

#### Children of survivors (G2 group)

**Fig. 3:**
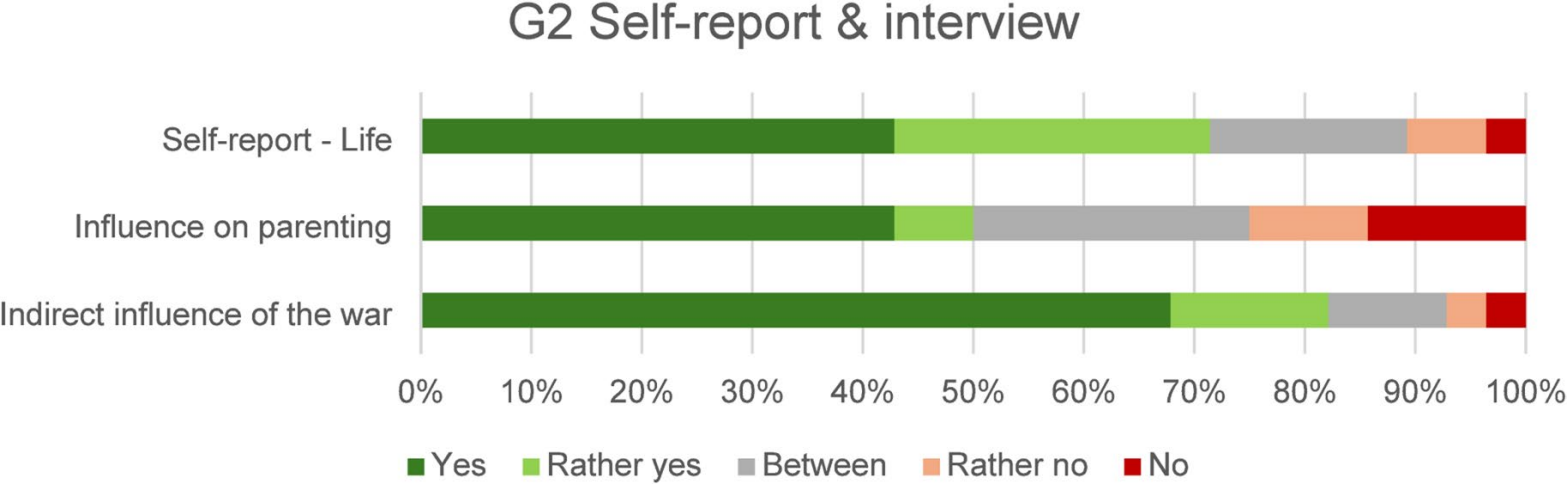
G2 responses in the self-report questionnaire and to interview questions.

In the self-report questionnaires (Self-report – life), 71% of G2 were satisfied with their personal life; the overall results are shown in Fig. 3.

Half of the participants answered yes or rather yes to the interview question *Do you think the war had an effect on the way your parents raised you?* (Influence on parenting). Several participants expanded this question to include their specific experience of how they had been affected by the war; responses included: sensitivity to the war, pride in parents and country, preoccupation with the topic of the war, fear for loved ones, over-vigilance or caution. To the question *Do you think the war had an impact on you*,* even though you did not experience it directly?* (indirect influence of the war), 82% answered yes/rather yes. Several participants expanded on the question by sharing their experiences. Most of them mentioned overprotection by their parents and trying to make sure they were well provided for as children and prepared for emergencies, sometimes even strict upbringing; increased vigilance towards social phenomena; emphasis on not taking what one has for granted; talking about the war – dividing the time before and after the war, but in most cases avoiding overly personal memories. The results for G2 are shown in Fig. 3.

#### Results summary

In G1, reduced grey matter volume was observed in the Crus II region of the cerebellum, as well as in the parahippocampal gyrus, fusiform gyrus, middle temporal gyrus, and postcentral gyrus compared to control group (CG). Psychological assessments indicated elevated symptoms of post-traumatic stress (PCL-5), decreased levels of life satisfaction (SWSL), and increased post-traumatic growth indicators (PTGI) in G1 compared to CG.

A significant increase in PTGI was found in G2 as compared to CG; no significant differences were found in grey matter volume, post-traumatic stress symptoms, or life satisfaction.

## Discussion

In this study, we investigated the long-term effects of war-related stress on structural changes in the brain’s grey matter and in psychological aspects in two generations of people who were directly or indirectly affected by the war in the countries of the former Yugoslavia.

### Survivors of conflicts in the former Yugoslavia (G1 group)

In this study, the most significant finding of the voxel-based morphometry analysis in G1 was a reduction in grey matter volume in the left cerebellar Crus II. Although the cerebellum has historically been associated primarily with motor functions^30^, recent research has confirmed its involvement in non-motor processes, including cognition, emotion, social cognition, and autobiographical memory^31,32^. The posterior lobe structure Crus II, part of the cerebellum’s phylogenetically newer regions, has been linked to social mentalisation, expression of self-related emotions, and the processing of autobiographical memory^33,34^. A recent meta-analysis identified a consistent association between PTSD and reduced volume in Crus II, with symptom severity showing a stronger correlation than diagnosis alone^35^. Functional MRI studies have also shown increased activation of Crus II during the recall of sad and negative autobiographical memories^33^. The posterior cerebellum is anatomically connected to paralimbic areas and contributes to the integration of emotion and behaviour^35^. A reduction in volume was also found in the right and left parahippocampus, part of the paralimbic system. The parahippocampal gyrus (PHG), located on the ventromedial surface of the temporal lobe and closely associated with the hippocampus, plays a role in emotional processing and memory consolidation^36^. Nardo and his team focused their study on grey matter concentration; they found a reduction in density in the PHG (and in the posterior cingulate and insular cortex) in PTSD subjects. They suggest that the altered function of PHG may lead to memory impairments seen in PTSD or impaired processing of emotional stimuli and integration of traumatic memories^37^. Also, reduction in the volume of this structure has been found in patients with PTSD^38^. In another work^39^, the authors argue that disrupted parahippocampal-prefrontal coupling may result in altered memory suppression in patients with PTSD^39^. A reduction in grey matter was also observed in the middle temporal gyrus (MTG), which is involved in cognition and memory processing, particularly in the retrieval and suppression of emotional memories, including PTSD flashbacks^40^. Volume reduction in the left MTG has been associated with impaired retrieval of autobiographical memories in PTSD^41^. The fusiform gyrus (FG), known for its role in face and body recognition and complex visual processing, is also involved in memory, emotional perception, and threat detection. Reduced volume in this region has been linked to deficits in visual and emotional processing of social stimuli in PTSD, potentially contributing to symptoms such as hypervigilance and social dysfunction^38,42,43^. Although the postcentral gyrus, which is primarily responsible for somatosensory processing, is not usually examined in research on psychological trauma, a study of war veterans with PTSD was conducted to examine somatosensory responses to non-threatening stimuli. Veterans with PTSD did not show typical responses to touch in the postcentral gyrus, superior parietal region, and right prefrontal cortex. Unlike participants with PTSD responded differently to non-threatening tactile stimuli This result suggests that PTSD manifests itself not only through psychological symptoms but also through somatosensory symptoms, such as altered responses to tactile stimuli^44^.

The finding that all regions in which we found volume reductions in G1 were associated with post-traumatic stress disorder or its symptoms corresponds with the results of the PCL-5 questionnaire, which revealed higher symptoms of persistent post-traumatic stress in G1. The threshold of 31 points or more^45^, on the basis of which a preliminary diagnosis of PTSD can be made, was exceeded in approximately half of the subjects in group G1. Therefore, while it cannot be conclusively stated that individuals in G1 meet the full diagnostic criteria for PTSD, they do exhibit pronounced symptoms indicative of post-traumatic stress. Furthermore, we found lower satisfaction with life (SWSL) in G1 as compared to CG. Similar results were found in an earlier study that examined individuals affected by the conflict in the former Yugoslavia 10 years later. These individuals still showed symptoms of post-traumatic stress decreased quality of life 10 years after the conflict^46^. Our results extend these findings to a broader time horizon. From our previous work examining Holocaust survivors^12^, we know that increased stress symptoms and lower well-being can persist over the long term and even throughout an entire lifespan. However, the same study found that post-traumatic growth also persists, as is the case with G1 in the current study. The coexistence of post-traumatic growth and stress is not an uncommon phenomenon. Some studies have explored the possibility that PTG is not a completed event based on an experienced event, but a process of seeking to cope with stress^47^.

Given that the reduction in grey matter volume in G1 occurred in the Crus II area, which is demonstrably involved in the processing of negative memories and social mentalisation, one plausible explanation for this reduction is the suppression of negative experiences, which may function as a coping mechanism in G1. This hypothesis is indirectly supported by interviews from our G2 participants, who are the children of war survivors, even though they are not direct descendants of our G1 participants. In interviews, G2 often reported that discussions of the war were common in their households. However, these conversations typically lacked deep introspection regarding the emotional experiences of their parents.

Empirical studies suggest that while thought suppression or avoidance may offer short-term relief, it is associated with the persistence of PTSD symptoms in the long term^48^. This finding is consistent with the psychological questionnaires used in this study, which indicate that G1 participants exhibit higher levels of PTSD symptoms and lower life satisfaction. However, results from the PTGI suggest that, despite these challenges, G1 participants also experience post-traumatic growth, reporting positive evaluations of their personal and professional lives. Many have achieved above-average incomes, high levels of education, and have successfully integrated into countries other than their homeland.

Although suppression is generally considered a maladaptive long-term coping strategy, it does not preclude the possibility of resilience. In their research on resilience in post-war Southeastern Europe, Kelmendi and Hamby identified emotional self-regulation as a prominent trauma coping mechanism characteristic of this region^49^. In G1, the coping mechanism appears to involve conscious or unconscious suppression of negative autobiographical memories, leading to the creation of a “protective shell” that allows them to cope with trauma and live a full life, without completely eliminating their emotional scars.

### Children of survivors (G2 group)

The second generation in this study consisted of people who were born after the end of the war to parents affected by the war (in Bosnia and Herzegovina or in Croatia) or the bombing in Serbia in 1999. Most participants grew up in the post-war countries of the former Yugoslavia, some were born elsewhere, and a few participants were born and spent their childhood in the Czech Republic, although they continued to have a relationship with their parents’ countries of origin. In the interviews, the participants reported that the topic of war is still alive. It was repeatedly mentioned that for some families, the war is a constant part of almost every conversation, and time is split into pre-war and post-war periods, with parents often talking about what happened in terms of their perspective of everyday life but sharing little about their inner experiences and feelings.

We found no differences between the brain structures of the G2 and their peers in CG2. Neither were differences found in the PCL-5 and SWSL questionnaires: participants do not show differences in persistent symptoms of post-traumatic stress nor feel lower satisfaction with life compared to the control group. Like G1, G2 do show higher post-traumatic growth than CG. However, participants from G2 did not have a significant difference in PCL-5, nor did they exceed the cut-off threshold for PCL-5 in greater numbers (only three subjects had scores above 31). Thus, it cannot be said that this group carried persistent post-traumatic stress.

The absence of post-traumatic stress and the presence of post-traumatic growth are not mutually exclusive. Although in most interviews, with the exception of two participants, participants in G2 rated the war as having had an impact on them, a possible explanation is that they have successfully integrated the difficulties they experienced into their lives, or that PTG has served as an adaptation process, as we indicated above for G1^47^.

Based on our recent research, we found that the second generation differed in some ways from the non-war-affected control group, but we could not determine whether this was due to the post-war environment^50^, family and collective history^51^, transgenerational transmission, or a combination of these factors. It seems that these questions will be the subject of new research in the future^52^.

## Limitations

Our group cannot represent an independent cross-section of the Balkan population. In our research, we only worked with individuals who volunteered for the study; their motivation to participate itself may have biased the sample. Furthermore, these people have an above-average socioeconomic background; G1 participants had lived in a country other than their home country for a long time.

G1 participants had experienced war in many forms and in different countries of the former Yugoslavia (Bosnia and Herzegovina, Croatia, Serbia). It is not a perfectly homogeneous group, but on the other hand, it is a model sample of people affected by war in various forms.

Our two stress groups, G1 and G2, are not parents and children. Therefore, we cannot observe direct transmission from parents to offspring.

## Conclusion

Individuals affected by the war in the former Yugoslavia exhibit reduced grey matter volume in brain structures associated with post-traumatic stress disorder (PTSD) symptoms and autobiographical memory processing. These individuals continue to experience persistent symptoms of post-traumatic stress and report lower life satisfaction compared to control participants. At the same time, they demonstrate higher levels of post-traumatic growth and, in self-reports, assess their lives positively. This pattern suggests the presence of a specific, albeit imperfect, coping mechanism that enables a more reactive response to perceived or actual threats, while still allowing for the pursuit of a fulfilling life. In contrast, the second generation, raised in a challenging post-war environment shaped by a pervasive war narrative, do not exhibit structural brain changes or significant differences in life satisfaction and stress symptoms compared to their peers in the control group. Although they do not show persistent symptoms of post-traumatic stress, they tend to perceive that the difficulties caused by the post-war environment have a negative impact on their lives. At the same time, they report increased post-traumatic growth, suggesting that they may have effectively integrated these challenges into their lives or that post-traumatic growth serves as an adaptive ongoing mechanism supporting their ability to cope with adversity.

Finally, these findings highlight the need for stress research to consider the role of the cerebellum, particularly Crus II, which has long been overlooked in studies of non-motor brain functions.

## Methods and analysis

### Research

The research was conducted between 2022 and 2025 at the Central European Institute of Technology—Research Centre of Masaryk University in Brno. It was approved by the Ethics Committee of Masaryk University in accordance with the Declaration of Helsinki. The ethics committee’s approval code number is EKV-2021-076. Informed consent was obtained from each participant.

This project represents an interdisciplinary research effort examining the psychological, physiological, and neuroimaging correlates of war-related stress in civilian populations. The current study specifically focuses on data pertinent to the assessment of brain structure, utilising structural MRI findings alongside psychological questionnaires and interview data.

### Recruitment

Volunteers were recruited between 2022 and 2024 through media reports, university information channels, social networks, and a lecture at the Lastavica club, which brings together the Balkan diaspora in the Czech Republic.

### Participants

We investigated participants who, as civilians, survived the war in former Yugoslavia and second-generation survivors - children of war survivors. The group of participants we categorised as the first generation (G1) was composed of people (*n* = 45) who experienced multiple war events and were under life-threatening stress during war activity in then-Yugoslavia in 1991–1995 (Bosna and Herzegovina, Croatia) or experienced NATO bombing in Serbia in 1999, or, in some cases, a combination of these events. To the question, *How did you survive the war? What did you experience during the war?* participants provided a variety of responses, including:

Participants reported experiencing a range of war-related events, which are described below in narrative form for clarity. These included shelling and bombing, during which individuals sought safety in cellars, shelters, or other hiding places. Many were forced to abandon or witnessed the destruction of their homes. Some sustained war-related injuries, and others experienced the death or injury of loved ones, or witnessed such events happen to others. Separation from family members and loved ones was frequently reported, along with a constant fear for their safety. Participants also described extreme shortages of basic necessities such as food, medicine, water, and electricity, as well as conditions of poverty. Some were subjected to discriminatory or humiliating behaviour by soldiers. In a few cases, individuals were forced into military service, although none of the participants were professional soldiers. Additionally, participants reported difficulties associated with forced emigration, including experiences of bullying or rejection, loss of social capital, and financial hardship. After the experience of the conflict, these people emigrated from their home country directly to the Czech Republic or to other countries and then to the Czech Republic, where they currently live.

The second generation (G2) contains people who are descendants of war survivors (*n* = 28) and were not exposed to war-threatening stress but grew up in a post-war environment. In addition to five persons of Czech nationality who were born in the Czech Republic to parents who had emigrated to the Czech Republic, there were participants of Bosnian, Croatian, Serbian and Montenegrin nationality who were temporarily in the Czech Republic as part of a study programme. G1 and G2 are not family members (with a few exceptions). G2 participants are not direct descendants of G1.

The control group (CG) consisted of Czechs or Slovaks living in Czechia who had no war experienc; the group were divided into two subgroups corresponding to G1 and G2, we called the subgroups CG1 and CG2 (*n* = 73; 45 + 28). Participants in the control group were unrelated to each other.

### Exclusion criteria

Brain impairment (brain injuries or diseases, tumours, neurodegenerative diseases), severe psychiatric disorders*(e.g. psychosis), significant cognitive decline (Before the examination itself, participants underwent a cognition test, a subtest from WAIS-III: Digit Span^53^ and completed an Intellectual Potential Test)^54^. Furthermore, participants with technical artefacts or excessive movement artefacts in the MR images were excluded.

* Due to the nature of the research, individuals who were previously diagnosed with post-traumatic stress disorder or believed they might be diagnosed with it were retained in the sample.

The stress groups and their assigned control groups did not differ significantly in age, sex, or education. Income was significantly higher in G1 than in CG1**. The education and income of G2 and CG2 were not assessed because most participants were students (one-third of respondents did not answer this question because they were not employed during their studies, while others only had part-time or temporary jobs.). Age was compared using the two-sample t-test; sex, education, and income were compared using the Fisher exact test (Table 3).

**As income was assessed only at the time of examination and does not reflect a long-term indicator of socioeconomic status, it was not included as a variable. The results of the statistical analysis including this variable are provided in the supplementary material to our manuscript (Figure S1, Table S1).

**Table 3:** Demographic data of the research population.

	G1	CG1	*p*-value
Count	45	45	-
Age	38 ± 9	37 ± 10	0.5870
Sex	23 M / 22 F	20 M / 25 F	0.6732
University education	29 Y / 16 N	32 Y / 13 F	0.6523
Income	30 H / 15 L	11 H / 34 L	**0.0001**

	G2	CG2	*p*-value
Count	28	28	-
Age	25 ± 3	24 ± 3	0.3255
Sex	9 M / 19 F	11 M / 18 F	0.7828

#### MRI

MR examinations were performed on a 3T scanner Siemens Prisma using a 64-channel head coil. The MRI protocol for voxel-based morphometry included 3D T1-weighted magnetisation prepared rapid gradient echo (MPRAGE) sequence with TR = 2.3 s, TE = 2.33 ms, TI = 0.9 s, FA = 8°, isometric voxel size 1 mm in FOV 224 × 224 mm and 240 slices.

Anatomical MRI data were analysed using SPM12 (www.fil.ion.ucl.ac.uk) and CAT12 toolbox (www.neuro.uni-jena.de/cat) running in Matlab R2020a.

The standard recommended pipeline was used for segmentation, in short: high resolution data were segmented into grey matter (GM) using the SPM Tissue Probability Map (TPM) and registered into common MNI space using shooting template IXI555_MNI152_GS. Finally, spatially normalised and modulated GM maps were smoothed with 6 mm FWHM isotropic Gaussian kernel.

Group-level statistics for stress effects were computed using a second-level model in SPM12. Modulated GM images were corrected for total intracranial volume and subsequently analysed. Two independent two-sample t-tests were performed to compare GMV maps between stress and control groups: one comparing G1 vs. CG1, and another comparing G2 vs. CG2. Sex and age were included as nuisance variables in both models.

Resultant t-statistic maps were initially thresholded at a P value of < 0.001 uncorrected and then only significant clusters at *P* < 0.05 FWE cluster level were picked.

#### Psychological questionnaires and self-report and interview

Participants completed a set of psychological questionnaires (Table 4), a self-assessment, and a semi-structured interview, allowing them to answer questions briefly or provide more detailed responses if they wished.

**Table 4:** Psychological questionnaires.

PTGI	Post-traumatic growth inventory	Assesses positive outcomes of experiencing stressful life events. The Inventory contains 21 items and measures 5 factors: New Possibilities, Relating to Others, Personal Strength, Spiritual Change, and Appreciation of Life. Participants were asked to mark the degree of perceived change (0 = I did not experience this change as a result of my crisis to 5 = I experienced this change to a very great degree). The score range is from 0 to 105, with higher scores indicative of greater post-traumatic growth^55^ The Posttraumatic Growth Inventory is a copyrighted instrument and was used for research purposes with permission.
PCL-5	The PTSD check list for DSM-5	A 20-item self-report instrument for measuring symptoms of PTSD based on DSM-5. Respondents are asked to consider a “list of problems and complaints that people sometimes have in response to stressful experiences” that is supposed to rate how much they “have been bothered by each problem in the past month”. Every item is scaled from 0 (“not at all”) to 4 (“extremely”). Completing the inventory takes approximately 5–10 min. The PLC-5 questionnaire can be evaluated in several ways. A cut-off score between 31 and 33 items is used for a provisional diagnosis of PTSD^45^
SWSL	Satisfaction with life scale	A 5-item self-report instrument. Respondent answers are marked on Likert scale ranging from 1 (“strongly disagree”) to 7 (“strongly agree”), and are presented in raw scores, with a total score range from 5 to 35. The SWLS focuses on cognitive aspects of life satisfaction rather than emotional; moreover, SWLS does not focus on specific domains. SWLS makes it possible to weigh different aspects of life based on personal criteria^56^
MSPSS	Multidimensional scale of perceived social support	A 12-item self-report scale. The instrument measures subjectively assessed social support based on three subscales addressing a different source of support: (a) family, (b) friends and (c) significant others. Respondent answers are marked on Likert scale ranging from 1 (“very strongly disagree”) to 7 (“very strongly agree”)^57^
	Brief COPE	A 28-item self-report inventory focusing on effective and ineffective ways to cope with stressful events. It is useful tool for detecting emotional responses to serious events e.g. health conditions, financial stress, mental illness, injuries and more. Respondent answers were marked on scale ranging from 1 (“I haven’t been doing this at all”) to 4 (“I’ve been doing this a lot”)^58^

The results of the psychological questionnaires were summarised using the median, as well as the Interquartile range (IQR). To test for statistical differences, the non-parametric Mann-Whitney U test was employed. The significance level for all statistical tests was set at *p* < 0.05.

### Self-report and interview

All participants completed a short self-assessment of their life and work achievements to date. Satisfaction with life and career was rated on a scale of yes, rather yes, somewhere in between, rather no, no. Participants from G2 did not rate career success as they were predominantly students. Stress groups (G1 and G2) additionally provided information on war experiences affecting themselves or their families through semi-structured interviews, forming the basis for a separate psychological study. For the current text, we report only demographic data, life satisfaction ratings, and war-related experiences. G1 participants were asked whether war was the worst event of their life, and how they coped; G2 participants were asked whether they felt that the war had affected them indirectly and whether the war had affected their parents’ parenting style.

## Supplementary Information

Below is the link to the electronic supplementary material.

Supplementary Material 1

## Data Availability

The datasets used and/or analysed during the current study available from the corresponding author on reasonable request.

## Abbreviations

G1: First generation
G2: Second generation
CG: Control group
CG1: Control group for G1
CG2: Control group for G2
MRI: Magnetic resonance imaging
PTSD: Post-traumatic stress disorder
ICD-11: International classification of diseases, 11th revision
PTGI: Post-traumatic growth inventory
PCL-5: PTSD checklist for DSM-5
SWSL: Satisfaction with life scale
MSPSS: Multidimensional scale of perceived social support
Brief-COPE: Brief coping orientation to problems experienced inventory
