# Supplementary material for: Long-term neurostructural and psychological effects of war stress in two generations of civilians from the former Yugoslavia
Source: Sci Rep. 2026 Mar 17;16:13878. doi: 10.1038/s41598-026-44241-w (PMC13128900; doi:10.1038/s41598-026-44241-w)
Supplement: Supplementary file 1 — Supplementary Material 1 [file 41598_2026_44241_MOESM1_ESM.docx]

**Supplementary material**:

Long-term neurostructural and psychological effects of war stress in two generations of civilians from the former Yugoslavia

Voxel-based morphometry analysis illustrating grey matter volume differences between survivors (G1) and the control group (CG1), with income included as a covariate (Figure S1 and Table S1).

**Figure S1**


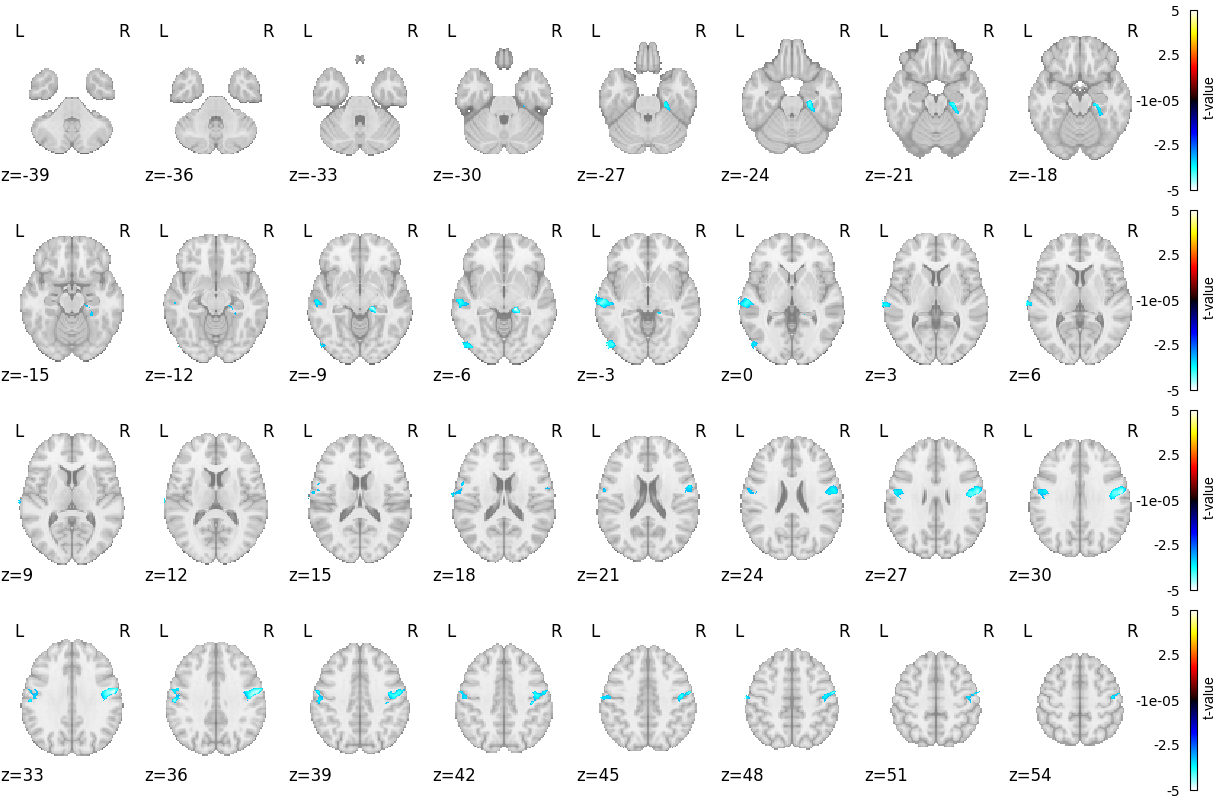


*Figure 1: Comparison of grey matter volume between survivors (G1 group) and relevant control group with income included covariates.*

**Table S1**

| **Laterality** | **Structures** | **p-corrected** | **Peak (MNI, mm)** | **Cluster Size (**cm^3^**)** |
| --- | --- | --- | --- | --- |
| L | Occipital Inferior | 0.0028 | -55.5;-78.5;-3.5 | 0.772 |
|  | Postcentral  Precentral | 0.0205 | -56.5;-18.5;36.5 | 2.596 |
|  | Temporal Middle | 0.0386 | -72.5;-20.5;6.5 | 2.130 |
| R | ParaHippocampal  Fusiform  Cerebelum 4 5 | 0.0095 | 16.5;-29.5;-7.5 | 1.826 |
|  | Postcentral  Precentral | 0.0002 | 56.5;-6.5;32.5 | 5.357 |

*Table S1: Five significant clusters after correction (on each row), where the grey matter volume in G1 is smaller than in CG1 with income included in covariates*
